# BMD Regulation on Mouse Distal Chromosome 1, Candidate Genes, and Response to Ovariectomy or Dietary Fat

**DOI:** 10.1002/jbmr.200

**Published:** 2010-08-04

**Authors:** Wesley G Beamer, Kathryn L Shultz, Harold F Coombs, Victoria E DeMambro, Laura G Reinholdt, Cheryl L Ackert-Bicknell, Ernesto Canalis, Clifford J Rosen, Leah Rae Donahue

**Affiliations:** The Jackson LaboratoryBar Harbor, ME, USA

**Keywords:** DISTAL CHROMOSOME 1, MOUSE CONGENIC STRAINS, BMD CANDIDATE GENES, OSTEOBLASTS, DIETARY FAT

## Abstract

The distal end of mouse chromosome 1 (Chr 1) harbors quantitative trait loci (QTLs) that regulate bone mineral density (BMD) and share conserved synteny with human chromosome 1q. The objective of this article was to map this mouse distal Chr 1 region and identify gene(s) responsible for BMD regulation in females. We used X-ray densitometry [ie, dual-energy X-ray Absorptiometry (DXA), micro–computed tomography (µCT), and peripheral quantitative computed tomography (pQCT)] to phenotype a set of nested congenic strains constructed from C57BL/6BmJ (B6/Bm) and C3H/HeJ (C3H) mice to map the region associated with the BMD QTL. The critical region has been reduced to an interval of 0.152 Mb that contributes to increased BMD when C3H alleles are present. Histomorphometry and osteoblast cultures indicated that increased osteoblast activity was associated with increased BMD in mouse strains with C3H alleles in this critical region. This region contains two genes, *Aim2*, which binds with cytoplasmic dsDNA and results in apoptosis, and *AC084073.22*, a predicted gene of unknown function. Ovariectomy induced bone loss in the B6/Bm progenitor and the three congenic strains regardless of the alleles present in the critical BMD region. High dietary fat treatment (thought to suppress distal Chr 1 QTL for BMD in mice) did not induce bone loss in the congenics carrying C3H alleles in the critical 0.152 Mb carrying the *AIM2* and *AC084073.22* genes. Gene expression studies in whole bone of key congenics showed differential expression of *AC084073.22* for strains carrying B6/Bm versus C3H alleles but not for *Aim2*. In conclusion, our data suggest that osteoblasts are the cellular target of gene action and that *AC084073.22* is the best candidate for female BMD regulation in the distal region of mouse Chr 1. © 2011 American Society for Bone and Mineral Research.

## Introduction

There is general recognition that skeletal traits are both environmentally and genetically complex. The genetic basis of osteoporosis results from alterations in many genes that individually make small contributions. The search for genes that contribute to the pathogenesis of osteoporosis has intensified as aging populations expand worldwide. Enhancing that search has been remarkable growth in knowledge of bone biology over the past two decades and equally remarkable advancements in techniques and analytical methods within the genetics field.

Over the past decade, investigations aimed at finding osteoporosis-related genes have adopted the genome-wide association approach using populations that have been phenotyped for bone mineral density (BMD) and densely genotyped. Intriguing findings have included the vitamin D receptor, the Sp1 binding site in *COL1A1*, and the estrogen receptor *ESR1*.(1) However, replication of the results among studies has been difficult for a variety of reasons (eg, population stratification, ethnicity, physiologic factors, and gender), and associations have been rather low. Nevertheless, a number of reports have shown that regions and specific genes on human chromosome 1 (Chr 1) are associated with BMD, a widely used marker of osteoporosis. For example, Reed and colleagues(2,3) found that absorptive hypercalciuria (genetic location 1q23-24) coupled with reduced spinal BMD yielded adenylate cyclase (*ADCY10*) as a candidate gene for osteoporosis. Quantitative trait loci (QTLs) located at 1q21-24 have been reported for BMD in lumbar spine by Koller and colleagues,(4) as well as by Deng and colleagues(5) and Wilson and colleagues(6) at 250 and 270 cM, respectively. Recently, Ioannidis and colleagues have reported a meta-analysis of nine genome-wide association studies limited to women that found several chromosomal regions, including 1p13.3–1q23.3, with significant BMD association.(7) In two additional studies, Schaffer and colleagues(8) found significant association of BMD with 1q23 in young 25- to 45-year-old Mexican Americans, and Ichikawa and colleagues(9) reported a significant association of BMD with a haplotype block containing 11 genes on 1q in premenopausal white women, but the specific gene was not identified.

Not surprisingly, expanding discoveries in biomolecules and their mechanisms of action affecting osteoblast or osteoclast functions have led to examination of specific genes for roles in osteoporosis. In a comprehensive review by Deng and colleagues, the number of specific bone-related genes with a plausible connection to osteoporosis is extensive, and these genes are located across the genome.(10) Examples of such genes have been described by (1) Chen and colleagues,(11) who reported significant correlation of *signal transducer and activator of transcription 1* (*STAT1*) upregulation with low BMD in white women, (2) Brochmann and colleagues,(12) who found that *Bone Morphogenetic Protein 2* (*BMP2*) appears to be regulated by *secreted phosphoprotein 24 kDa* (*SPP24*), (3) Reppe and colleagues,(13) who found eight genes, including *chromosome 1 open reading frame 61* (*C1orf61*), that in transiliac biopsies were significantly associated with BMD in postmenopausal white women, and (4) Mori and colleagues,(14) who reported a significant association of *thrombospondin* (*ADAMST4*) with spinal and femoral BMD in Japanese women. Each of these four genes is located on mouse Chr 1.

We and others have reported that the distal region of mouse Chr 1, which shares conserved synteny with the long arm (q) of human Chr 1, carries QTLs that regulate BMD.(14,15) To capitalize on the control of experimental variables and the ease of gene mapping in mice, we developed a set of nested congenic strains that have allowed us to test which regions on Chr 1 are responsible for regulation of BMD.(16) Using these nested congenic strains, we found that the *BMD5* QTL on Chr 1 consists of three regions with sex- and site-specific bone-compartment effects.(14) Here we present fine mapping of one of these regions designated QTL1. We also present additional phenotyping data with dietary manipulation and initial expression data for two BMD candidate genes, *absent in melanoma 2* (*Aim2*) and *AC084073.22*.

## Materials and Methods

### Mice

The inbred mouse strains used for the studies reported herein were obtained from our research colonies at The Jackson Laboratory (Bar Harbor, Maine, USA). The C57BL/6BmJ (B6/Bm) and B6.C3H Chr 1 congenic sublines of mice were produced by pair matings, with progeny weaned at 22 to 25 days of age and housed in groups of 2 to 5 of the same sex in polycarbonate cages (324 cm^2^) with sterilized white pine shavings. Colony environmental conditions included 14:10-hour light/dark cycles with free access to acidified water (pH 2.5, with HCl to retard bacterial growth) that contains 0.4 mg/mL of vitamin K (menadione Na bisulfite) and an irradiated NIH 31 diet containing 6% fat, 19% protein, and Ca:P of 1.15:0.85, plus vitamin and mineral fortification (Purina Mills International, Brentwood, MO, USA). All animal studies were approved by the Institutional Animal Care and Use Committee of The Jackson Laboratory.

### Dietary treatment

At 2 months of age, female mice were assigned to experimental groups, with group sizes of 16 to 27 mice. Mice were fed either a high-fat diet (42% fat, 1% cholesterol, 0.5% cholate, 20% protein, and Ca:P ratio of 1.08:0.85; hereafter 42% fat diet) or continued on the standard NIH 31 (6% fat) diet described earlier. Dietary treatments were continued until mice were 4 months of age. Consumption studies demonstrated that mice consumed equivalent amounts of food by weight (high-fat/cholesterol/cholate diet, 3.13 g/mouse/day; NIH 31 diet, 3.22 g/mouse/day).

### Congenic sublines and genotyping

**Fig. 1:**
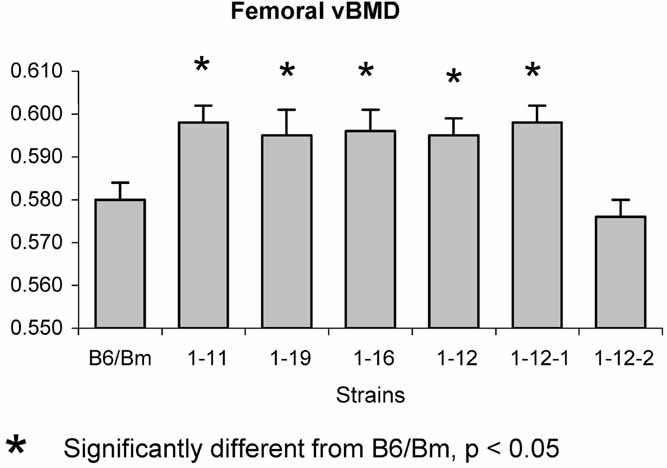
Femoral pQCT vBMD for 16-week-old females from B6/Bm and six congenic strains covering distal chromosome 1. Congenic means that differ from B6/Bm are indicated by asterisks. Data are means ± SEM (group size *n* = 21 to 48). The formal designations for these inbred strains are 1-11: B6/Bm.C3-(*D1Mit111-D1Mit359*)BmJ; 1-19: B6/Bm.C3-(*D1Mit111-rs6197487*)BmJ; 1-16: B6/Bm.C3-(*D1kls17-1-rs3654101*)BmJ; 1-12: B6/Bm.C3-(*D1Mit355-D1Mit359*)/BmJ; 1-12-1: B6/Bm.C3-(*D1Mit355-kls2-1*)/BmJ; and 1-12-2: B6/Bm.C3-(*rs31592036-D1Mit359*)/BmJ.

We previously reported B6.C3H-1 congenic strains for distal mouse Chr 1 that carry femoral volumetric BMD (vBMD) and trabecular microstructure regulation in female and male mice.(14,16) The genetic segment of interest, designated QTL1, was mapped to *D1Mit355.* To fine map this genetic segment, two new congenic sublines were developed, B6.C3H-1-12-1 (1-12-1) and B6.C3H-1-12-2 (1-12-2), by mating B6.C3H-1-12 (1-12) N_10_ generation mice with B6/Bm progenitor strain mice. The N_11_F_1_ offspring were intercrossed to obtain segregating N_11_F_2_ progeny that were genotyped to identify smaller C3H segments. These were used to establish 1-12-1 and 1-12-2 sublines that are homozygous for different overlapping segments of the C3H genome on distal Chr 1. The formal nomenclature for the congenic strains is found in the legend to Fig. 1.

Mice were genotyped by preparing genomic DNA from digestion of 1-mm tail tips in 0.5 mL of 50 mM NaOH for 10 minutes at 95°C; then the pH was adjusted to 8.0 with 1 M Tris-HCl. Genotyping of individual mouse DNAs was accomplished by polymerase chain reaction (PCR) using oligonucleotide primer pairs from several sources (Research Genetics, Birmingham, AL, USA; Invitrogen, Carlsbad, CA, USA; IDT, Coralville, IA, USA; and Qiagen, Valencia, CA, USA). These primer pairs amplify sequence-length polymorphisms (SSLPs) consisting of CA repeat sequences wherein the length of the repeat is strain-specific (Mit Markers, www-genome.wi.mit.edu/cgi-bin/mouse/index). Details of standard PCR reaction conditions have been provided previously.(17) PCR products from B6/Bm, C3H, and F_1_ hybrids were used as electrophoretic standards in every gel to identify the genotypes of mice (ie, *b6/b6*, *b6/c3h*, *c3h/c3h*). In addition to the SSLP markers just described, five additional markers were developed in our laboratory to further define the congenic regions. Specifically, “novel” SSLPs were identified in the region of interest, and primers were designed to amplify these regions by PCR. Eight markers representing flanking sequences for CA repeats were designed with the aid of Frodo.wi.mit.edu/cgi-bin/primer3/ and MacVector. Custom primer pairs were obtained from Invitrogen and tested against B6/Bm and C3H genomic DNA to confirm polymorphisms (listed in Table 1). In addition, 14 single-nucleotide polymorphisms (SNPs) within the 171.1 to 179.3 region (NCBI Ensembl Build 37, Release 56) that were assayed for the sublines are presented in this article. SNP genotyping was performed at KBiosciences (Herts, UK; www.KBioscience.co.uk).

**Table 1:** Primer Sequences

Gene	Forward primer	Reverse primer
*AC084073.22*	TGTACACAAAGAGCAGGAC	CTCACAGTACAGTTTGGTAAATTG
*Aim2*	CACCCTCATGGACCTACACTA	CGTTGTTAGTAAATCAGCAGTTCT
Primer name		
*kls27-3*	TTTATTGAGGAAATTGACAGAA	GTGTCATGGGAAGTTTCATT
*kls17-1*	AAGGGGGTTCTACCTATCAG	CTTCCCATTTGACCACTAAA
*kls5-1*	GAATATGGAATCTGACTGTGG	ATGAAAACCTGATCTTGGTG
*rs30595455*	ATTTGTGGTTCCAGGCAACATC	GGGACTCGGGACTTTTTGAATG
*rs30707811*	AGGACATTATTAGCAACTCGCCC	AGATTCACCTGCCCTACTTTACTCC
*kls8-4*	TCAAGCCTCAACTAAAGGTC	GTTTTGCCTTGTGTTTCTTC
*rs3718078*	GATGTCCTTGGGGTTCAAATGAC	TGCTATCAGGCTCGCTTAGTTCAG
*rs31606438*	TGCTGGGTTCATTTCAAAGGTC	ATGGGGTAGGGTAGTTATGTCGG
*rs6197487*	TCTCAGGTTATTTTCAGGCTTG	AAGAGTCCCAGACACTTCTCCCTC
*rs31335968*	ATTTCTGTAGGCTCCAGGGGTGTC	TAGGGTAGTTATTGCGGGGACG
*rs31592036*	TATCCCCCTGTGACTTGCTGAC	TACCCTCCAGTGAATGCTGCCTTC
*kls4-6*	AGTCAGCACCACTGTCATTT	TGGCTTTGTACCAATACCAT
*kls9-2*	TCCATGGAGTGAGTCTAACC	TCTTCTACAGGGCAGGACTA
*kls4-5*	GTTCTGCTCCATCTTCCTTT	GAAGGATTGGAATTTTGACA
*rs3699295*	TCCATAACTCCAGACACCTTTGC	AACCCATAGCCAGCCCAGATACTG
*kls2-1*	GGCTTGGATAATGATGAATG	CCATCCATCTCTCCACTTAG
*rs3655994*	TGCCATGAATTACCACAATCCC	GCAGCATAGCTTCGCTGTTTG

### Necropsy

At 16 weeks of age, when mice have acquired their adult femoral mass,(18) females from the B6/Bm progenitors and the newly developed distal Chr 1 sublines were necropsied and whole-body weights recorded. Whole-body areal BMD (aBMD) and body-composition data were collected by PIXImus (DEXA; GE-Lunar, Madison, WI, USA) densitometry, as described below. Skeletal preparations (lumbar vertebral columns, pelvis, and attached hind limbs) were placed in 95% ethanol (EtOH) for a period of not less than 2 weeks. Lumbar vertebral columns, tibias, and femurs were dissected free of remaining muscle and connective tissue and placed in 95% EtOH for storage. vBMD was assessed by peripheral quantitative computed tomography (pQCT) as described below.

### Ovariectomy

Four-month-old B6/Bm and congenic strain mice were treated by sham surgery or ovariectomy under isofluorane anesthesia and aseptic conditions. Postoperatively, the mice were maintained under standard dietary (NIH 31 diet) and environmental conditions for 6 weeks. Necropsies were performed and whole-body densitometry data obtained by PIXImus (procedure described below).

### X-ray densitometry

#### PIXImus for aBMD

Female mice were measured at necropsy for whole-body aBMD and percent body fat using the PIXImus dual-energy X-ray absorptiometer (GE-Lunar). The PIXImus was calibrated daily with a mouse phantom provided by the manufacturer. Mice were placed ventral side down with each limb and tail positioned away from the body. Full-body scans were obtained and X-ray absorptiometry data gathered and processed with manufacturer-supplied software (Lunar PIXImus 2, Version 2.1, GE-Lunar). The head was specifically excluded from all analyses owing to concentrated mineral in skull and teeth.

#### pQCT for vBMD bone densitometry

vBMD was measured on the left femur of female B6/Bm and congenic subline mice, as described previously.(14) Briefly, isolated femur lengths were measured with digital calipers (Stoelting, Wood Dale, IL, USA). Femurs were measured for density using the SA Plus densitometer (Orthometrics, White Plains, NY, USA). Calibration of the SA Plus instrument was accomplished with a manufacturer-supplied phantom. The bone scans were analyzed with Orthometrics software Version 5.50. Mineral content was determined with thresholds such that mineral from most partial voxels (0.07 mm) would be included in the analysis. Isolated femurs were scanned at seven locations at 2-mm intervals beginning 0.8 mm from the distal ends of the epiphyseal condyles. Given the variation in femur lengths, the femoral head could not be scanned at the same location for each bone and thus was not included in the final data. Total vBMD values were calculated by dividing the total mineral content by the total bone volume and expressing the result in milligrams per square millimeter. Cortical thickness was obtained at the midshaft scan.

#### µCT40 for distal trabecular bone

Trabecular bone components of the distal femur were measured by µCT (MicroCT40, Scanco Medical AG, Bassersdorf, Switzerland) as described previously.(14) The MicroCT40 unit was calibrated weekly with a phantom standard provided by Scanco. The femurs were scanned at the energy level of 55 KeV and intensity of 177 µA, followed by analysis of the trabecular bone (BV) with Scanco software Version 5.0. Approximately 150 cross-sectional slices were made at 12-µm intervals at the distal end. Beginning at the edge of the growth plate and extending in a proximal direction, 100 contiguous slices were selected for analysis.

### Quantitative Real-Time PCR

RNA was extracted from femurs of four 10-week-old congenic and B6/Bm female mice fed the respective diets for 2 weeks. Femurs were isolated and snap frozen in liquid nitrogen, RNA then was isolated using the Total RNA Isolation System (Promega, Madison, WI, USA) as per the manufacturer's instructions. DNA was removed using DNase treatment and removal reagents (Ambion, Inc., Austin, TX, USA). RNA quality and quantity were assessed using an Agilent Bioanalyzer (Caliper Technologies Corp., Hopkinton, MA, USA). Then 400 ng of RNA was converted to cDNA in a Real-Time (RT) reaction using the MessageSensor RT Kit (Ambion, Inc.) and random decamers as primers. The RT-PCR primers for *Aim2* and *AC084073.22* were designed and purchased from PrimerDesign Ltd. (Southampton, UK). The cDNA then was diluted 1:10 with water. For each PCR reaction, 2.5 µL of diluted cDNA was added to 5 µL of Power SYBR Green Master Mix (Applied Biosystems, Foster City, CA, USA) and 100 nM of each forward and reverse primer and 1.5 µL of H_2_O in a total reaction volume of 10 µL. Cycling conditions were a hold of 2 minutes at 50°C, a hold of 10 minutes at 95°C, and 40 cycles at 95°C for 15 seconds and 60°C for 1 minute, and all reactions were run on the ABI 7500 Sequence Detection System (Applied Biosystems). Expression of *Aim* and *AC084073.22* was assessed by comparing the expression of each with an average of the housekeeping genes *Gadph* and *B2M* using the ΔΔ*C*_*t*_ method.(19) Probe efficiencies were optimized by PrimerDesign (http://primerdesign.co.uk/research_with_integrity.html). Each experiment was run in triplicate with different cDNA preps from the same mice. Primer sequences for *Aim2* and *AC084073.22* are reported in Table 1.

### Primary calvarial osteoblast cultures

Calvarial osteoblasts were isolated using a modified method from Wong and Cohn.(20) Calvaria were dissected from neonatal mice (1 to 3 days of age) and maintained in sterile phosphate-buffered saline (PBS) until the dissection was completed. Calvaria were subjected to four sequential 15-minute digestions in an enzyme solution containing 0.05% trypsin (Invitrogen) and 3.6 U/mL of collagenase P (Roche Diagnostics, Indianapolis, IN, USA) at 37°C on a rocker platform. Cell digestion fractions 2 to 4 were collected and chilled by the addition of cold Dulbecco's Modified Eagle Medium (DMEM) containing 10% fetal bovine serum (FBS), 100 U/mL of penicillin, and 100 µg/mL of streptomycin. Fractions were pooled, centrifuged, and resuspended in DMEM with 10% FBS. Cells were plated at a density of 1.5 × 10^4^ cells/cm^2^ in 35-mm dishes. After 7 days of culture, some dishes were changed to differentiation medium (α-MEM, 10% FBS, 50 mg/mL ascorbic acid, and 8 mM β-glycerophosphate), whereas other cultures were stained for alkaline phosphatase. On day 18 of culture, remaining dishes were stained with alizarin red for calcium deposits.

Alzarin red–stained wells were imaged using a Leica Wild M10 stereomicrocsope and a Leica DFC 300 FX cooled CCD (Wetzlar, Germany). Images were processed using ImageJ imaging software (rsbweb.nih.gov/ij/). Specifically, a threshold was established to remove background staining, applied to each 0.5× image, and then the number of pixels corresponding to alzarin red–stained areas (histogram intensity of 0 to 80) was determined. Total alzarin red^+^ pixels from these images (one image per 35-mm culture well) representing six independent cultures were averaged for B6/Bm and 1-12 congenics.

### Histomorphometry

Groups of B6/Bm and 1-12 congenic females were dual labeled for bone histomorphometric analyses. Mice were injected with 20 mg/kg of calcein (intraperitoneal) at 15 weeks and 50 mg/kg of demeclocycline 7 days later. Mice were necropsied 48 hours following the demeclocycline injection. Femurs were dissected free of muscle and fixed in 10% NBF at 4°C, then stored in 70% EtOH at room temperature. The femur samples were embedded undecalcified in methyl methacrylate. Longitudinal sections 5 µm thick were cut on a Micron microtome (Micron, Richard-Allan Scientific, Kalmazoo, MI, USA) and stained with 0.1% toluidine blue (pH 6.4). Static parameters of bone formation and resorption were measured in a defined area between 360 and 1440 µm from the growth plate using an Osteomeasure Morphometry System (Osteometrics, Atlanta, GA, USA). For dynamic histomorphometry, mineralizing surface per bone surface and mineral apposition rate were measured in unstained sections under ultraviolet light, as described previously.(21) Bone-formation rate was calculated. The terminology and units used are those recommended by the Histomorphometry Nomenclature Committee of the American Society for Bone and Mineral Research.(22)

### Statistical assessment

Statistical evaluation was undertaken using JMP Version 7.0 (SAS, Inc., Cary, NC, USA). For the pQCT and µCT data, any body size differences between strains were accounted for by using body weight and femur length as covariates when they contributed significantly to an overall ANCOVA model.(23) Data are expressed as adjusted least squares mean ± SEM in all figures. Differences between means for B6/Bm and each congenic subline were tested by Student's *t* test, with significance declared when a *p* ≤ .05 was observed.

## Results

**Fig. 2:**
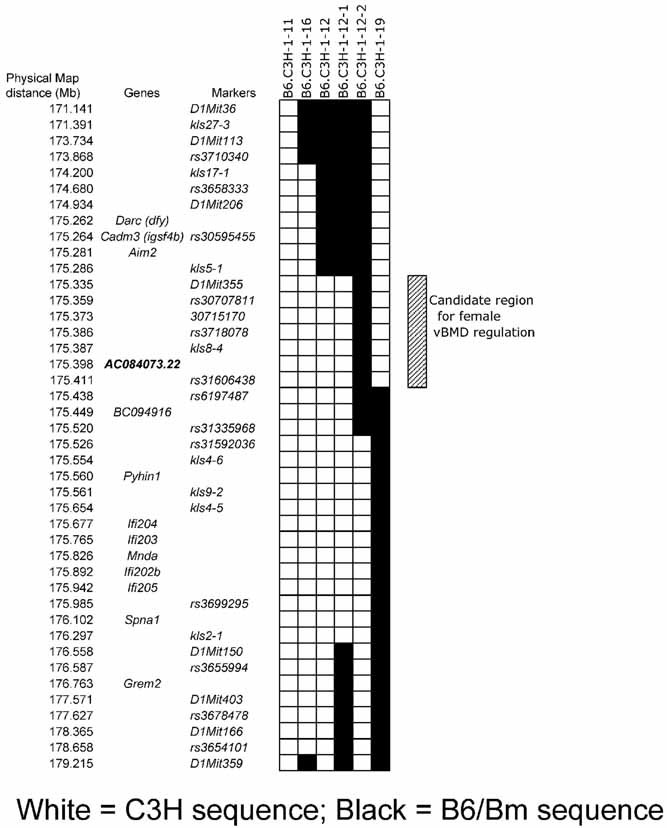
Genetic haplotype map of distal chromosome 1 for six congenic sublines. Physical locations, genetic markers, and selected genes are given for each subline, along with diagrammatic presentation of the alleles in the chromosomal region represented in the sublines. The rectangle to the right of the diagram indicates the critical region containing the vBMD regulation.

The phenotypic femoral vBMD data obtained from 4-month-old B6/Bm progenitor and congenic strain females are presented in Fig. 1. Congenics B6.C3H-1-11. B6.C3H-1-19, B6.C3H-1-16, and B6.C3H-1-12 (hereafter 1-12) all have significantly greater vBMD values than B6/Bm, as reported previously.(14) To further refine the Chr 1 critical region, two new strains, 1-12-1 and 1-12-2, were developed from 1-12. vBMD in 1-12-1 is significantly increased compared with B6/Bm, whereas vBMD in 1-12-2 is not different from that in B6/Bm. The congenic strains that demonstrate increased vBMD all have C3H alleles for the shared critical 0.152 Mb critical region located between markers *kls5-1* and *rs6197487*, whereas the 1-12-2 congenic, which does not have increased femoral vBMD, has B6/Bm alleles in this critical region (Fig. 2).

Distal femurs from female B6/Bm progenitor and 1-12 congenic mice were examined by histomorphometry for evidence of increased bone formation. The data in Table 2 showed that in comparison with B6/Bm bones, the 1-12 congenic bones had (1) significantly increased bone volume owing to an increase in trabecular thickness, (2) an unchanged trabecular number, (3) an increased mineralizing surface, and (4) an increased bone-formation rate. These data are consistent with our previous observations of the B6.C3H-1-11 strain, the progenitor of the 1-12 strain.(14) The histomorphometric data did not reveal differences in the number of osteoblasts, osteoclasts, or eroded surface/bone surface (latter data not shown).

**Table 2:** Histomorphometric Data From Distal Femurs of B6/Bm and B6.C3H-1-12 Females at 16 Weeks of Age

	BV/TV (%)	Tb.Th (µm)	Nob/Tar (/mm^2^)	Nob/BPm (/mm)	Noc/Tar (/mm^2^)	Noc/BPm (/mm)	Tb.Sp (µm)	Tb.N (/mm)	Ms/Bs (%)	MAR (µm/day)	BFR/B.Sd (µm^3^/µm^2^/day)
B6/Bm											
Mean	6.96	26.70	84.09	20.56	7.92	1.90	361.78	2.60	7.44	0.989	0.074
SEM	± 0.37	± 0.63	± 4.80	± 0.62	± 0.46	± 0.16	± 13.82	± 0.10	± 0.48	± 0.033	± 0.006
B6.C3H-1-12											
Mean	8.82	29.72	89.48	19.43	8.61	1.88	315.04	2.95	8.74	1.093	0.096
SEM	± 0.61	± 0.84	± 3.77	± 0.69	± 0.44	± 0.11	± 15.52	±0.16	± 0.37	± 0.045	± 0.006
*p*	.02	.01					.04		.047		.02

**Fig. 3:**
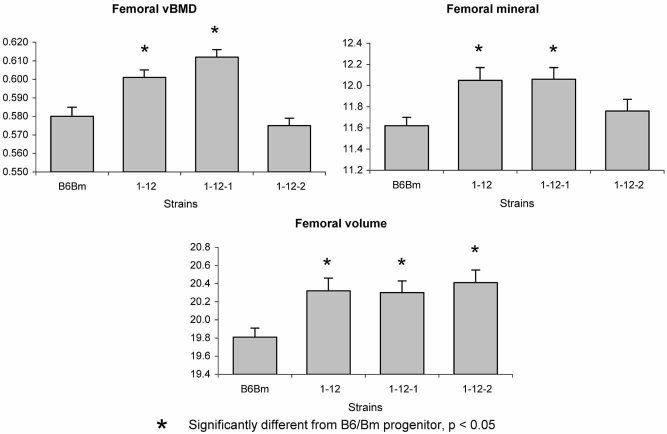
Femoral pQCT data for B6/Bm progenitor, 1-12, 1-12-1, and 1-12-2 congenic strain females at 16 weeks of age. Significant differences compared with B6/Bm mice are indicated by asterisks. Data are means ± SEM (group size *n* = 36 to 48).

Of the two congenic strains carrying smaller C3H genomic segments from 1-12, 1-12-1, but not 1-12-2, showed increased femoral vBMD with respect to B6/Bm. To determine whether changes in vBMD are a consequence of changes in total mineral or in total volume, femurs from B6/Bm, 1-12, 1-12-1, and 1-12-2 females were examined by pQCT. The pQCT data in Fig. 3 showed that the greater vBMD correlated with increased mineral content. Interestingly, all three congenic strains showed increased femoral volume compared with B6/Bm.

**Fig. 4:**
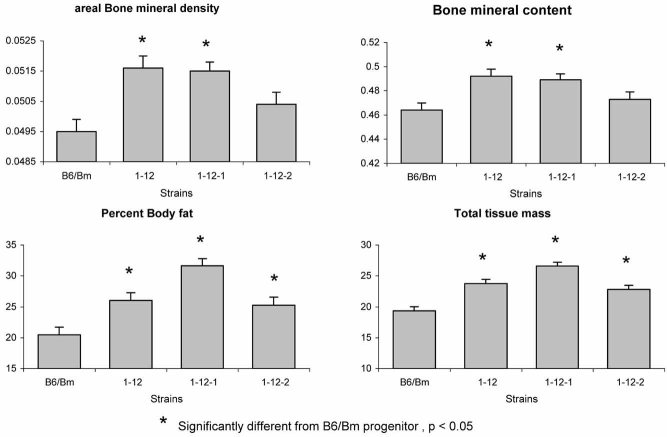
Whole-body data for B6/Bm progenitor, 1-12, 1-12-1, and 1-12-2 congenic strain females at 16 weeks of age. Significant differences compared with B6/Bm mice are indicated by asterisks. Data are means ± SEM (group size *n* = 19 to 21).

Whole-body densitometry by PIXImus was employed to determine whether the increased vBMD and mineral content observed in the femur were detectable in the entire skeleton. Figure 4 presents the aBMD, bone mineral content (BMC), percent body fat, and total tissue mass for B6/Bm, 1-12, 1-12-1, and 1-12-2 congenic strain females. The aBMD and BMC in 1-12 and 1-12-1 mice were significantly greater than in either B6/Bm or 1-12-2 mice. The percent body fat and total tissue mass of all three congenic strains were significantly greater than in the B6/Bm progenitor strain. No differences were found in total-bone area of any strain (data not presented).

**Fig. 5:**
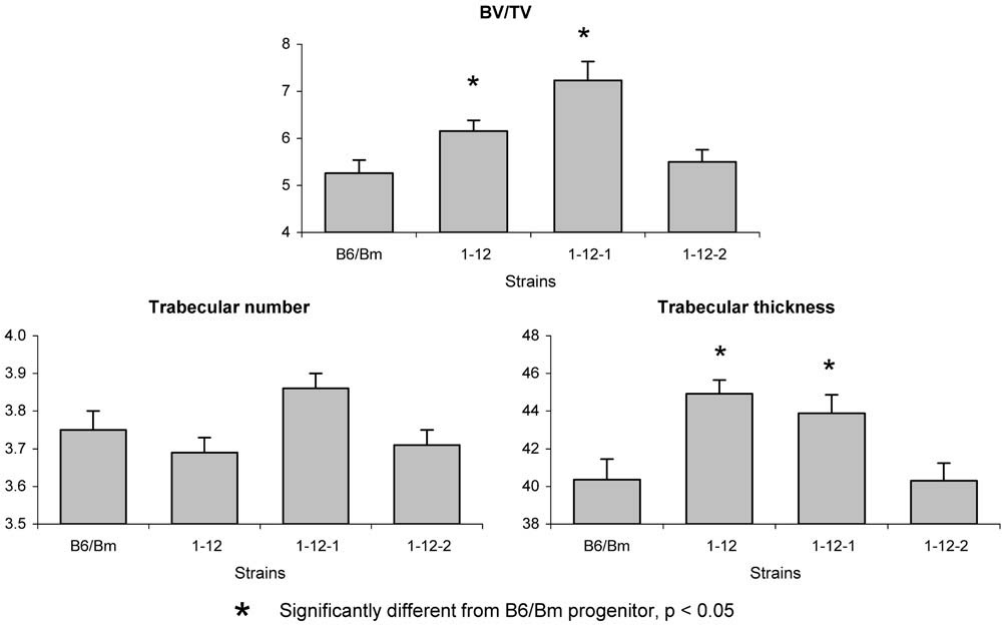
µCT data from distal femurs of 1-12, 1-12-1, and 1-12-2 congenic strain females at 16 weeks of age. Significant differences among strains are indicated by asterisks. Data are means ± SEM (group size *n* =19 to 20).

Microstructural properties of trabecular bone in the distal femurs of B6/Bm, 1-12, 1-12-1, and 1-12-2 strains were assessed by µCT (Fig. 5). The 1-12 and 1-12-1 congenics had significantly increased bone volume fraction (BV/TV) and trabecular thickness compared with either B6/Bm or 1-12-2 femurs. Trabecular number did not differ among strains.

**Fig. 6:**
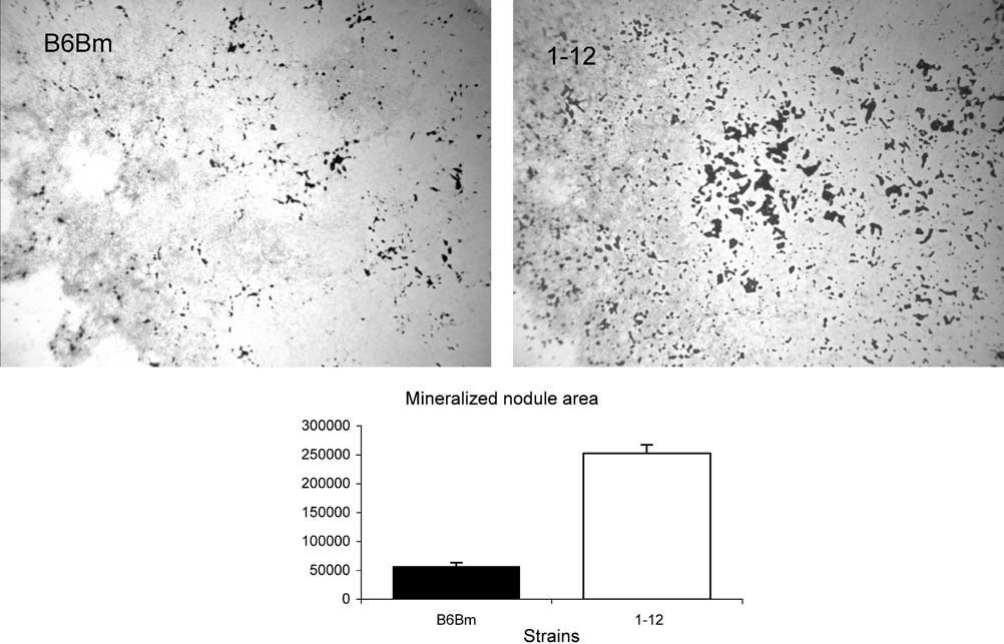
B6/Bm progenitor and 1-12 neonatal calvarial osteoblasts on day 18 of culture stained for alizarin red. Digitized images (2300 × 1700 µm) from 6 wells per genotype were used to quantify the alizarin red–stained nodules.

To gain insight into the biology underlying the differences in mineral content and vBMD observed between the congenic lines, we established primary cultures of neonatal calvarial osteoblasts from 1-12 and B6/Bm mice (Fig. 6). The alizarin red staining of cultures indicated that there was 4.4-fold increase in mineralized nodule area in 1-12 osteoblasts on day 18 compared with B6/Bm osteoblasts, suggesting that osteoblast progenitors and activity are greater than observed for B6/Bm mice.

### Bone density responses to ovariectomy or dietary fat

**Fig. 7:**
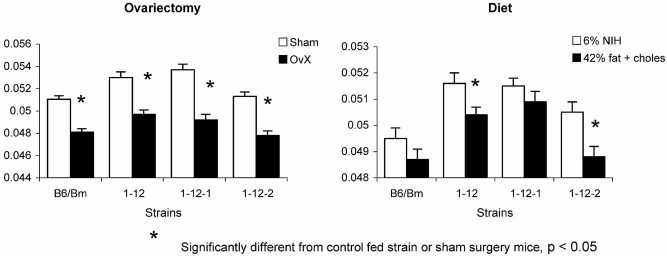
Whole-body data for B6/Bm, 1-12, 1-12-1, and 1-12-2 congenic strains 6 weeks after ovariectomy or after 8 weeks of treatment with 6% fat NIH 31 diet or 42% fat diet. Significant treatment-induced differences within strains are indicated by asterisks. Data are means ± SEM (group size *n* = 16 to 27).

The B6/Bm and congenic strains were tested for their skeletal responses to regulatory challenges in the form of gonadectomy and dietary fat manipulation. Loss of ovarian steroid for 6 weeks resulted in all strains losing significant amounts of aBMD, as shown in Fig. 7. The aBMD data presented in Fig. 7 showed that 1-12 and 1-12-2 strains lost significant aBMD, whereas the B6/Bm and 1-12-1 mice did not have significant bone loss.

### Gene expression

According to the current mouse genome assembly (Ensembl Release 58, May 2010, www.ensembl.org), the 0.152-Mb critical region defined by our nested congenic strains contains two known genes, *absent in melanoma 2* (*Aim2,* ENSMUSG00000037860) and a putative protein coding sequence listed in the Ensembl mouse genome assembly as *AC084073.22* (ENSMUSG00000037849). We examined the expression of these two genes in 1-12-1 and 1-12-2 mice by quantitative real-time PCR (qRT-PCR) analysis of whole-bone cDNA to determine if genotype differences existed. The expression data for congenic strain female mice at 10 weeks of age are presented in Table 3. The results showed that both 1-12-1 and 1-12-2 female mice expressed higher amounts of *Aim2* than similarly aged B6/Bm female progenitor mice on the standard 6% fat NIH 31 diet.

**Table 3:** Expression of *Aim2* and *AC084073.22* in Whole Femurs From Females

Strain	Gene	Fold change	*p* Value
B6.C3H-1-12-1	*Aim2*	2.11	.007
B6.C3H-1-12-2	*Aim2*	1.78	.005
B6.C3H-1-12-1	*AC084073.22*	0.55	.028
B6.C3H-1-12-2	*AC084073.22*	1.92	.049

*Note:* Fold change determined from comparison with B6/Bm controls.

On the other hand, comparison of expression for *AC084073.22* in 1-12-1 and 1-12-2 female mice treated with the 6% fat NIH 31 diet showed genotype differences. The 1-12-1 bone showed significantly less whereas 1-12-2 showed significantly more expression of *AC084073.22* than B6/Bm bone.

## Discussion

### Fine mapping and candidate gene analysis

Using new congenic sublines, we confirmed the female vBMD regulation associated with the *D1Mit355* region, as originally reported.(14) The two new congenic sublines, 1-12-1 and 1-12-2, yield a smaller genetic segment amenable for molecular and functional studies of candidate genes. Use of flanking markers (*kis5-1* to *rs6197487*) established the maximum size of the critical region to be 0.174 Mb. Current mouse genome annotation (Ensembl, Version 55) predicts only two genes in this region, *Aim2* and *AC084073.22*. These genes are members of the P200 cluster of interferon-activated genes(24) that now consists of 9 loci (www.ensembl.org/Mus). Meiotic recombination yielded congenic strain 1-12-1, which contains C3H sequences for *Aim2*, *AC084073.22*, and *BC094916*, whereas congenic strain 1-12-2 contains C3H sequences for *Pyhin1*, *Ifi204*, *Ifi203*, *Mnda*, *Ifi202a*, and *Ifi205*. The *BC094916* gene has been eliminated from the critical region by the increased BMD phenotype of the B6.C3H-1-19 strain, which does not carry the C3H allele for *BC094916*.

The mouse and human P200 cluster of interferon-activated genes is located on mouse distal Chr 1 (between 94.0 and 95.4 cM) and on human Chr 1 (between 1q21 and 23). The mouse region has approximately 9 paralogous genes (*Aim2*, *AC084073*, *BC094916*, *Pyhrin1*, *Ifi204*, *Ifi 203*, *Mnda*, *Ifi202b*, and *Ifi205*), whereas the homologous human Chr 1 region has 4 paralogues (*Aim2*, *Pyrin1*, *Ifi16* (*Ifi205*), and *MNDA*). In both species, these genes are sufficiently similar in structure to have arisen from duplication events and are involved with cell growth and differentiation. Collectively, the proteins derived from these genes carry all or part of the PAAD/DAPIN/pyrin domains though to be involved in apoptotic and inflammatory signaling (reviewed in ref. 24).

Functions have been described for several of the genes in mice, with *Ifi202* and *Ifi204* of particular interest for bone biology because they have been shown to support chondrogenic and osteogenic differentiation, respectively.(25,26) *Aim2* was thought originally to be a tumor-suppressor gene associated with human melanoma(27) and breast cancer cells.(28) More recently, *Aim2* has been considered to be a protein that recognizes and binds to cytosolic dsDNA, forming a capase-1-activating complex that leads to apoptosis of the cell.(29) *Aim 2* has 12 exons that encode for a protein of 354 aa. *AC084073* does not yet appear to have an identified counterpart in the human P200 cluster. The homologue may not have been discovered yet, or the mouse P200 cluster may have undergone considerably more gene duplication than that of the human. *AC084073* has 3 exons and predicted protein of 150 aa (www.ensembl.org). Its function remains to be described.

### Aim2

The complete genomic sequence for the B6/Bm allele of *Aim2* is available from the NCBI m37 mouse assembly. The complete genomic sequence for the C3H allele of *Aim2* is available from the Sanger mouse genomes project (www.sanger.ac.uk/modelorgs/mousegenomes/). There is one SNP in exon 2 (*rs31606638*) of C3H that is synonymous to B6/Bm, and there are at least 14 SNPs in the 3' UTR. All the SNPs in the 3' UTR potentially could influence transcript stability or translation. Binding of miRNAs or RNA-binding proteins to target sequences in 3' UTRs has been shown to influence translation.(30) For example, Wang and colleagues showed that a risk-conferring polymorphism for Parkinson disease in human populations resides in the 3' UTR for *FGF20*. The polymorphism is in a binding site for miRNA-433 and increases translation of *FGF20* both in vitro and in vivo. We used TargetScanMouse(31) to scan the *Aim2* 3' UTR for miRNA binding sites, compared these sites with the positions of the 3'-UTR SNPs, and found that three of the 3'-UTR SNPs reside in miRNA target sites. One, *rs47500792*, is in a putative binding site for mmu-miR335-3p, and the other two, *rs47154027* and *rs46439487*, are adjacent SNPs that are in a putative binding site for mmu-miR370. These potentially could influence the translation of *Aim2*; therefore, future work will be directed at testing the influence of these SNPs on *Aim2* translation.

*Aim2* is expressed in bone marrow and mature B cells,(32) which places expression in the area of the bone niche where preosteoblasts and preosteoclasts reside. *Aim2* has a demonstrated role in apoptosis by binding cytoplasmic dsDNA fragments.(29,33) When *Aim2* was tested by RT-PCR, we found that compared with B6/Bm progenitors, there was significant upregulation in both 1-12-1 and 1-12-2 mice fed the 6% fat NIH 31 diet compared with similarly treated B6/Bm controls. The 42% fat diet suppressed these expression differences in both strains. The whole-body PIXImus BMD measurement of 1-12-2, but not 1-12-1, mice was reduced by the 42% fat diet. Since 1-12-1 and 1-12-2 congenics carry different alleles for *Aim2* and have different constitutive BMD values, the similarity of expression level under control and experimental conditions would suggest that differential regulation of *Aim2* is not likely to be involved in BMD regulation.

### AC084073.22

The complete genomic sequence for the B6/Bm allele of *AC084073.22* is available from Ensembl Release 58. In addition, the complete genomic sequence for the C3H allele of *AC084073.22* is available from the Sanger Mouse Genomes project (www.sanger.ac.uk/modelorgs/mousegenomes/). In these data, there are 73 SNPs in the *AC084073.22* genomic region (which includes both introns and exons), and among these, there are three coding SNPs and one splice-site SNP. One of the three exonic SNPs is a nonsynonymous coding SNP. This is a known SNP (*rs31605595*) that results in an asparagine substitution for lysine in the coded protein. The other 2 exonic SNPs are in the 3' UTR, and using TargetScanMouse,(31) we found that neither of these SNPs overlaps with known miRNA binding sites. The splice-site SNP is also known (*rs31605596)* and resides in the 5' splice donor site of intron 1. In this case, the C3H variant maintains the consensus splice donor sequence (GUPuAGU), and the B6/Bm allele is nonconsensus (GUPuAGC). We found that compared with B6/Bm mice on the 6% fat NIH 31 diet, *AC084073.22* expression in bone was significantly decreased in 1-12-1 mice (which contain the C3H allele) but significantly increased in 1-12-2 mice (which contain the B6/Bm allele). The prediction based on these data is that alternative splicing of the B6/Bm allele could lead to upregulation of *AC084073.22*. On the other hand, the substitution of a lysine for an asparagine could have a deleterious impact on protein function. We used a tool available from the Panther database (www.pantherdb.org/tools/csnpScore.do) to calculate a subPSEC score based on an evolutionary analysis of this coding SNP.(34) The calculated subPSEC score was –1.6, which corresponds to an approximately 19% probability of being deleterious. Based on these data, we predict that the splice-site SNP is more likely to contribute to differential function of this gene in B6/Bm and C3H. More detailed analysis is needed to determine whether the noncanonical splice site in B6/Bm leads to a strain-specific transcript isoform and/or whether aberrant splicing underlies the differential expression we observed by quantitative RT-PCR. However, our data show that differential expression of this gene, and not *Aim2*, is correlated with the bone density phenotypes described herein. Therefore, we hypothesize that *AC084073.22* underlies the bone density regulation in females that we have mapped to distal Chr 1.

### Dietary fat and distal Chr 1 BMD regulation

By using a B6C3F2 cross and a 6% fat NIH 31 diet, we originally found that a region on distal Chr 1 increased BMD.(14) On the other hand, Farber and colleagues(35) did not observe a distal Chr 1 BMD QTL in B6C3F2 mice fed a 42% fat diet. Our congenic strain analyses identified a 0.152-Mb region of C3H sequences on distal Chr 1 shared by 1-12 and 1-12-1 strains that consistently influenced BMD. We hypothesized that the 42% fat diet would reduce the BMD phenotype associated with the 0.152-Mb region identified in our congenic mice. In fact, the aBMD value of the 1-12-1 congenic strain was not reduced by the 42% fat diet, whereas the aBMD values of the 1-12 and 1-12-2 strains were reduced by 42% fat diet. These data suggest that the genomic region shared by the 1-12 and 1-12-2 strains, but not by the 1-12-1 strain, carries a dietary fat-responsive locus that is different from the region shared by the 1-12 and 1-12-1 strains that regulates BMD. Although the hypothesis about dietary regulation of BMD in distal Chr 1 appears tenable, the actual candidate region for dietary action appears to reside in the 3.007-Mb region between *kls2-1* (176.297 Mb) and *rs3668273* (179.304 Mb).

The distal region of Chr 1 has a growing list of genes linked to bone regulation. For example, the *Darc* gene has been identified previously as regulating BMD in B6.CAST-1 congenic mice.(36) *Darc* is located at 175.262 Mb, which lies proximal and outside the C3H sequence found in 1-12 mice. A second gene is *Ifi204*, with a known role in osteoblasts. Specifically, the Ifi204 protein is part of a regulatory transcriptional complex and serves as a linker between Rb and Cbfa1 proteins, leading to expression of *osteocalcin* and *alkaline phosphatase* genes.(37) This gene lies within the 1.272-Mb region of the C3H sequence shared by 1-12-1 and 1-12-2 strains. As noted earlier, *Ifi202* has been reported recently to have a role in chondrogenesis.(25) The region of 1-12-2 mice that does not overlap with 1-12-1 mice contains about 78 genes that could be targets for high-fat-diet regulation. One of these genes is *Grem2*, whose expression was reported in fat by Farber and colleagues to be highly correlated with BMD in their B6C3F2 mice. We did not find an effect of diet on *Grem2* expression in bone for either 1-12-1 or 1-12-2 mice compared with B6/Bm mice (data not presented). *Grem2* has been shown to be an inhibitor of osteoblast differentiation through suppression of bone morphogenetic protein 2 (BMP-2) action, and its suppression promotes osteogenesis in vitro.(38) Our data suggest that *AC084073.22* is a fifth gene on distal Chr 1 with bone regulatory action.

There are several limitations to our study. First, the data reported herein apply only to female mice from these congenic strains. Second, we still do not know which polymorphic region is responsible for differences in *AC084073.22* expression, nor the precise mechanism responsible for changing the magnitude or direction of transcript expression. The AC084073.22 protein levels are not yet measureable to assess observed genotypic effects between 1-12-1 and 1-12-2 strains in any tissue. Third, we cannot exclude the possibility that there is a cell-autonomous defect in osteoclasts in 12-1 mice even though by histomorphometry ES/BS and number of osteoclasts per bone surface of 1-12 did not differ from B6/Bm. The absence of bone loss in 1-12-1 mice in response to a high-fat diet and the greater trabecular thickness by µCTof this subline could be due in part to impaired osteoclast activation or differentiation. Challenge studies with parathyroid hormone (PTH) could help to determine if the higher bone mass in the 1-12-1 congenic strain is due to increased bone formation alone or a combination of reduced bone resorption and increased formation. Finally, the *AC084073.22* gene is reported to be a member of the interferon (IFN)–activated P200 gene cluster based on its genetic sequence. At this time, there are no data demonstrating that either IFN-α or IFN-β change *AC084073.22* expression. Hence the relationship of *AC084073.22* to these cytokines remains to be determined. Nonetheless, our mapping and gene-expression data suggest that *AC084073.22* is a strong candidate gene for a bone regulatory QTL on distal Chr 1 that underlies BMD differences between B6/Bm and C3H mice. Understanding the mechanism of action of this gene and its relationship to cells in the bone remodeling unit will be important steps in our discovery process.
